# Deletion of Clock Gene *Period 2* (*Per2*) in Astrocytes Shortens Clock Period but Does Not Affect Light-Mediated Phase Shifts in Mice

**DOI:** 10.3390/clockssleep7030037

**Published:** 2025-07-17

**Authors:** Soha A. Hassan, Katrin S. Wendrich, Urs Albrecht

**Affiliations:** 1 Department of Biology, University of Fribourg, 1700 Fribourg, Switzerland; soha.hassan@suezuniv.edu.eg (S.A.H.); katrin.wendrich@aofoundation.org (K.S.W.); 2 Department of Zoology, Faculty of Science, Suez University, Suez 43518, Egypt

**Keywords:** light, resetting, phase shift, entrainment, advance, delay, *Per2* knockout

## Abstract

The circadian clock is a self-sustaining oscillator with a period of approximately 24 h, enabling organisms to anticipate daily recurring events, such as sunrise and sunset. Since the circadian period is not exactly 24 h and the environmental day length varies throughout the year, the clock must be periodically reset to align an organism’s physiology with the natural light/dark cycle. This synchronization, known as entrainment, is primarily regulated by nocturnal light, which can be replicated in laboratory settings using a 15 min light pulse (LP) and by assessing locomotor activity. An LP during the early part of the dark phase delays the onset of locomotor activity, resulting in a phase delay, whereas an LP in the late dark phase advances activity onset, causing a phase advance. The clock gene *Period 2* (*Per2*) plays a key role in this process. To investigate its contributions, we examined the effects of *Per2* deletion in neurons versus astrocytes using glia-specific *GPer2* (*Per2*/*GfapCre*) knockout (KO) and neuronal-specific *NPer2*KO (*Per2*/*NesCre*) mice. All groups were subjected to Aschoff type II protocol, where an LP was applied at ZT14 or ZT22 and the animals were released into constant darkness. As control, no LP was applied. Phase shift, period, amplitude, total activity count, and rhythm instability were assessed. Our findings revealed that mice lacking *Per2* in neurons (*NPer2*) exhibited smaller phase delays and larger phase advances compared to control animals. In contrast, mice with *Per2* deletion specifically in glial cells including astrocytes (*GPer2*) displayed normal clock resetting. Interestingly, the absence of *Per2* in either of the cell types resulted in a shorter circadian period compared to control animals. These results suggest that astrocytic *Per2* is important for maintaining the circadian period but is not required for phase adaptation to light stimuli.

## 1. Introduction

The mammalian circadian system is an intricate internal timekeeping system that synchronizes physiological and behavioral functions with the 24 h environmental daily cycle. The master oscillator in mammals is the hypothalamic suprachiasmatic nuclei (SCN), which are responsible for the coordination of central and peripheral clocks. The SCN are aligned with environmental cycles such as the day/night cycle by cues known as “Zeitgebers” [1,2]. The most prominent Zeitgeber is light.

Photoreceptors in the retina detect light and relay the photic signal to the SCN, which transmit this signal to other central and peripheral oscillators. The molecular mechanism of the circadian oscillator is made up of a transcriptional/translational feedback loop. This process begins when the BMAL1 and CLOCK proteins (i.e., positive elements of the loop that activate the transcriptional/translational feedback loop) form a heterodimer (CLOCK:BMAL1) that drives the transcription of the negative elements of the loop, namely, *Period* (*Per1*, *Per2*, and *Per3*) and *Cryptochrome* genes (*Cry1* and *Cry2*), leading to the expression of their respective proteins (PER, CRY). These negative elements, in turn, form their own heterodimer complex (PER:CRY) that suppresses their own transcription by inhibiting the activity of the CLOCK:BMAL1 complex [3,4].

In nocturnal rodents, nocturnal light signals can reset the circadian oscillator in the SCN, which leads to phase shifts of the circadian locomotor activity rhythm [5,6,7]. The responsiveness to light in nocturnal rodents is time-dependent. Wild-type (*WT*) animals exhibit phase delays when photic signals are applied early in the night and phase advances when signals are applied late at night, while no significant effect is observed when light is applied at midday or midnight [7].

The expression of the molecular clock genes *Per1* and *Per2* has been reported to be induced by light within the SCN and retina [8,9]. This modulation underscores their role in the light transduction pathway, light responsiveness, and subsequent re-entrainment of circadian rhythms.

*Per2* is a critical component of the molecular clockwork in the SCN of mammals, playing a pivotal role in resetting the circadian clock in response to light. Studies have shown that mutant *Per2* mice, as well as total and neuronal-specific *Per2* knockout mice, are unable to delay the clock when exposed to a light pulse at early night (ZT14), unlike their wild-type (*WT*) counterparts [10,11]. Furthermore, mutant *Per2* mice and total *Per2* knockout mice display a shorter period compared to their controls with a transition to arrhythmic circadian activity under prolonged constant darkness [12,13]. Notably, the shorter period length is also observed in neuronal-specific *Per2* knockout mice [11]. Interestingly, recent findings indicate that *Per2* also participates in the CREB pathway, contributing to the activation of the *Per1* gene in response to photic signals [14].

Circadian molecular clock genes are expressed not only in neurons of the SCN but also in various glial cells, including astrocytes [15,16]. Emerging evidence suggests that a functional and intact circadian molecular clockwork in astrocytes plays an essential role in regulating SCN outputs and behavioral rhythms—such as activity patterns and sleep homeostasis—in a manner that is autonomous and independent from neuronal clocks [17,18,19].

Astrocytic clock genes are reported to regulate the fluctuating expression of glutamate, ATP, and adenosine, which are crucial in controlling circadian system entrainment to photic cues and the rhythmic sleep/wake cycle [20,21]. Disruption of the astrocytic clock has been shown to alter various animal behaviors [22]. For instance, targeted deletion of *Per2* in astrocytes decreases anxiety-like and depression-related behaviors [13]. Furthermore, specific deletion of *Bmal1* in astrocytes leads to disruptions in cognitive functions and circadian locomotor activity rhythms in mice, resulting in longer period lengths compared to control groups [18,23].

Despite these findings, the role of the astrocytic *Per2* gene in regulating the re-entrainment of the circadian clock in response to photic signals remains unexplored. Therefore, the current study aims to investigate the potential role of the astrocytic *Per2* gene in the photic signal transduction pathway and circadian clock resetting in mice. This investigation will utilize astrocyte-specific *Per2* knockout mice (*GPer2*) and neuron-specific *Per2* knockout mice (*NPer2*) to compare the function of *Per2* in astrocytes in circadian periodicity and phase resetting with its function in neurons.

## 2. Results

### 2.1. Effect of Constant Darkness (DD) Without LP on Circadian Locomotor Activity Rhythm

**Figure 1:**
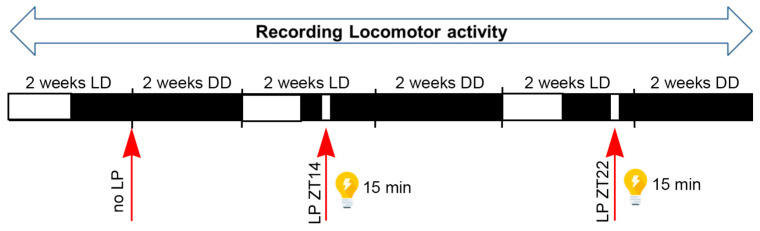
Experimental design for applying light pulse (LP) using the Aschoff type II protocol. For the 1st experimental condition, mice were kept in a light/dark cycle (LD) for 2 weeks and then released into constant darkness (DD) without LP for approximately 2 weeks. For the 2nd experimental condition, mice readapted to the LD cycle for 2 weeks and then were released into DD with an LP at ZT14 (early night) for 15 min on the last LD night. Subsequently, they were released into DD for approximately 2 weeks. For the 3rd experimental condition, the previous step was repeated but with an LP at ZT22 (late night). During the whole experiment, locomotor activity was recorded. ZT: Zeitgeber time. White and black bars indicate light and dark phases, respectively.

To determine the potential role of the *Per2* gene in the light response process, we used glia-specific *GPer2* (*Per2*/*GfapCre*) knockout (KO) [13] and neuronal-specific *NPer2* (*Per2*/*NesCre*) KO [11] mice. Glial and neuronal *Per2* deletion was verified by genotyping, immunohistochemistry, and Western blot techniques in brain tissues of mice [11,13]. These animals were compared with their corresponding cre-line *GfapCre* (*Gcre*) [24], *NesCre* (*Ncre*) [25], and littermate wild-type (*WT*) mice as control groups. The protocol is outlined in Figure 1 (see the Materials and Methods Section). We used an Aschoff type II protocol for the assessment of light-induced phase shifts [26,27].

**Figure 2:**
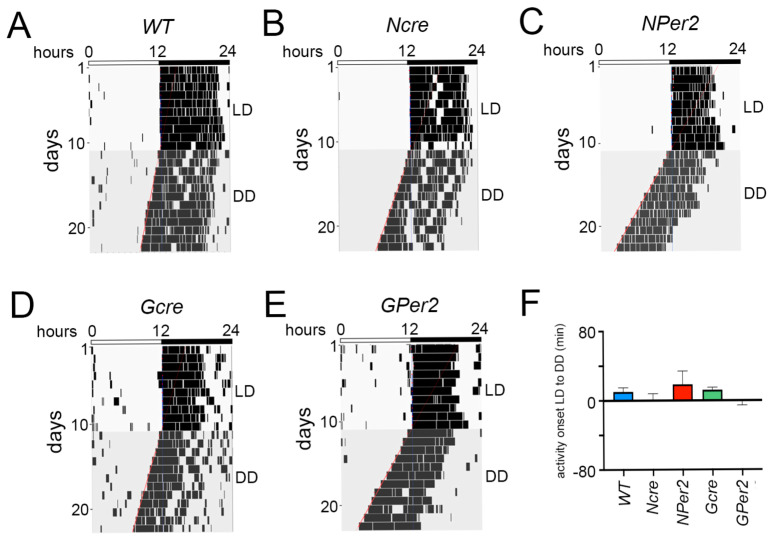
Representative actograms of wheel-running activity from mice receiving no light pulse. (**A**) Wild-type (*WT*), (**B**) nestin-cre (*Ncre*) control animals, (**C**) neuronal *Per2* KO (*NPer2*) mice, (**D**) gfap-cre (*Gcre*) control animals, and (**E**) glial *Per2* KO (*GPer2*) mice. (**F**) Quantification of the activity onsets at the LD to DD transition. Mice were kept in a light/dark cycle (LD; 12:12 h) and then released into constant darkness (DD) without a light pulse (LP) after the end of the last dark phase of the LD. White and black bars on top of each actogram indicate light and dark phases, respectively. The red lines indicate activity onset in DD. The blue lines represent activity onset in LD condition. Data are presented as mean ± SEM, *n* = 11 in all genotypes except for *NPer2* (*n* = 8). No differences between genotypes were observed.

**Figure 3:**
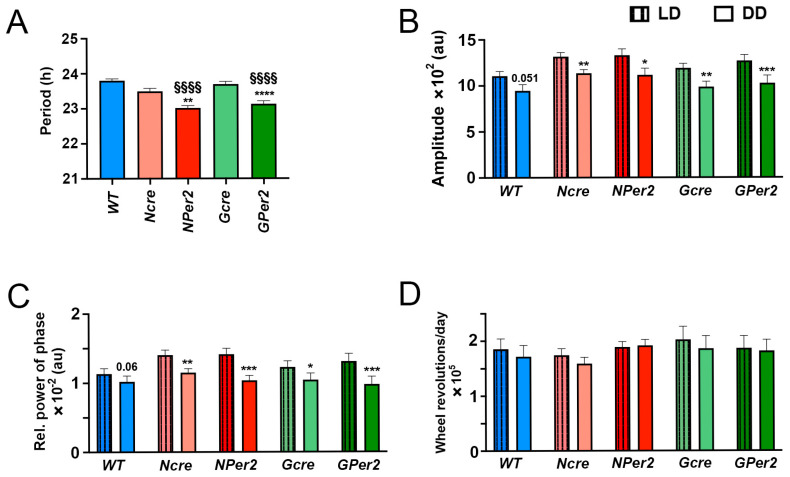
Effect of constant darkness (DD) without light pulse (LP) on locomotor activity parameters. (**A**) Period length: *WT* = 23.8 h ± 0.05, *Ncre* = 23.49 h ± 0.09, *NPer2* = 23.01 h ± 0.07, *Gcre* = 23.69 h ± 0.08 and *GPer2* = 23.1 h ± 0.09. One-way ANOVA: ^§§§§^ *p* < 0.0001 for *WT* animals, and ** *p* < 0.01 and **** *p* < 0.0001 to corresponding cre-control animals. (**B**) Amplitude, (**C**) relative power of phase, and (**D**) total activity counts were analyzed in *WT* (blue), *Ncre* (rose), *NPer2* (red), *Gcre* (light green), and *GPer2* (green) animals under LD (striped columns) and DD (plain columns) conditions. Paired *t*-test in (**B**–**D**): * *p* < 0.05, ** *p* < 0.01, *** *p* < 0.001 indicate differences between LD and DD conditions of each genotype. Data are presented as mean ± SEM, *n* = 11 in all genotypes except for *NPer2* (*n* = 8).

To determine the possible effects of transition from a light/dark cycle (LD) to constant darkness (DD) in the different genotypes, the circadian locomotor activity parameters were analyzed. Transition from LD to DD did not elicit significant differences in activity onsets between the genotypes (*p* > 0.05) (Figure 2A–F). Interestingly, the periods of *NPer2* and *GPer2* animals were significantly shorter compared to *WT*, *Ncre*, and *Gcre* control mice (Figure 3A), which is in line with previous observations made for *Per2* mutant and total *Per2* KO animals [11,12,28]. Comparison of the amplitude between LD and DD conditions revealed that for all genotypes amplitude was significantly shortened in DD, except for *WT* (*p* = 0.051) (Figure 3B). In LD, both *Ncre* and *NPer2* had a higher amplitude compared to *WT*; however, this was not significantly different (*p* = 0.07 and *p* = 0.08, respectively). No differences in the amplitude were observed between other genotypes (*p* > 0.05).

Next, we looked at the relative power of phase (FFT), a parameter used to indicate rhythm stability. Transition from LD to DD exhibited a significant decrease in FFT (indicating more rhythm instability) in all experimental groups, except *WT* (*p*= 0.06) (Figure 3C). However, the transition to DD did not affect the total locomotor activity counts in all investigated groups (*p* > 0.05, Figure 3D).

### 2.2. Effect of Neuronal and Astrocytic Per2 Deletion on Light Responsiveness and Circadian Clock Resetting

To investigate the possible effect of neuronal and astrocytic *Per2* in light responsiveness and circadian clock resetting, all genotypes were subject to a light pulse (LP) either at ZT14 (early night) or at ZT22 (late night) (Figure 1). Phase shift and other locomotor activity parameters were analyzed.

**Figure 4:**
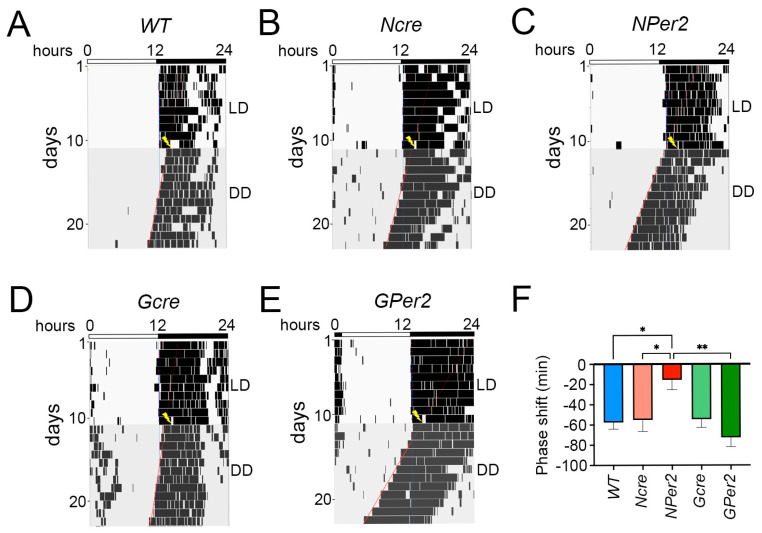
Representative actograms of wheel-running locomotor activity from mice receiving a light pulse (LP) at ZT14. (**A**) Wild-type (*WT*), (**B**) nestin-cre (*Ncre*) control animals, (**C**) neuronal *Per2* KO (*NPer2*) mice, (**D**) gfap-cre (*Gcre*) control animals, and (**E**) glial *Per2* KO (*GPer2*) mice. Mice were kept in a light/dark cycle (LD; 12:12 h) and received an LP of 15 min duration at ZT14 (yellow flash). Subsequently, they were released into constant darkness (DD). White and black bars on top of each actogram indicate light and dark phases, respectively. The red lines indicate activity onset in DD. The blue lines represent activity onset in LD condition. (**F**) Quantification of phase shifts after an LP at ZT14, with the following number of animals: *WT* and *Gcre*: *n* = 11 animals/genotype, and *Ncre*, *NPer2*, and *GPer2*: *n* = 8 animals/genotype. Data are presented as mean ± SEM. One-way ANOVA: * *p* < 0.05; ** *p* < 0.01 indicate differences between genotypes.

When an LP was applied at ZT14, the three control groups (i.e., *WT * (blue), *Ncre* (rose), and *Gcre* (light green), Figure 4F) delayed activity approximately by 1 h (−57.6 ± 6.6 min, −55.1 ± 11.3 min, and −54.6 ± 8.1 min, respectively) with no significant differences observed between the three groups (*p* > 0.05). On the other hand, *NPer2* displayed a significantly shorter phase delay (−15.5 ± 9.8) compared to the controls, *WT* and *Ncre* (*p* < 0.05). In contrast, *GPer2* showed a normal phase delay (−72.6 ± 9.2) with a tendency to be longer but not significantly different compared to its controls, WT and *Gcre* (*p* > 0.05). In addition, both KO groups showed a highly significant different phase shift (*p* < 0.01, Figure 4A–F).

**Figure 5:**
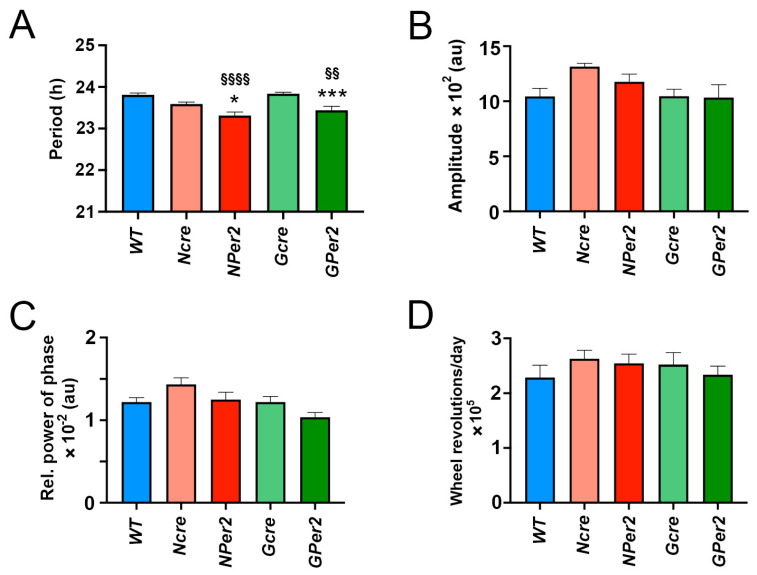
Effect of a light pulse (LP) at ZT14 on locomotor activity parameters. (**A**) Period length, (**B**) amplitude, (**C**) relative power of phase, and (**D**) total activity counts were analyzed in *WT* (blue), *Ncre* (rose), *NPer2* (red), *Gcre* (light green), and *GPer2* (green) animals in constant darkness (DD) after 15 min LP at ZT14. Data are presented as mean ± SEM, with n = 11 animals/genotype for *WT* and *Gcre*, and n = 8 animals/genotype for *Ncre*, *NPer2*, and *GPer2*. One-way ANOVA: ^§§^ *p* < 0.01; ^§§§§^ *p* < 0.0001 indicate differences between *NPer2* and *GPer2*, for *WT* animals. * *p* < 0.05; *** *p* < 0.001 indicate differences between *NPer2* and *GPer2* to their corresponding cre-control animals.

After an LP at ZT14 followed by a release into constant darkness, control groups showed shorter period length than 24 h (*WT*: 23.8 h ± 0.05, *Ncre*: 23.6 h ± 0.05, and *Gcre*: 23.8 h ± 0.04) and they were not significantly different (*p* > 0.05) (Figure 5A). Compared to their controls, *NPer2* and *GPer2* exhibited significantly shorter period length (*NPer2*: 23.3 h ± 0.09, *p* < 0.0001 vs. *WT* and *p* < 0.05 vs. *Ncre*; *GPer2*: 23.4 h ± 0.1, *p* < 0.01 vs. *WT* and *p* < 0.001 vs. *Gcre*). Both groups showed non-significant period lengths among them (*p* > 0.05, Figure 5A). In addition, the LP at ZT14 did not elicit any significant changes in amplitude, FFT, and total activity counts between all investigated genotypes (*p* > 0.05, Figure 5B–D).

**Figure 6:**
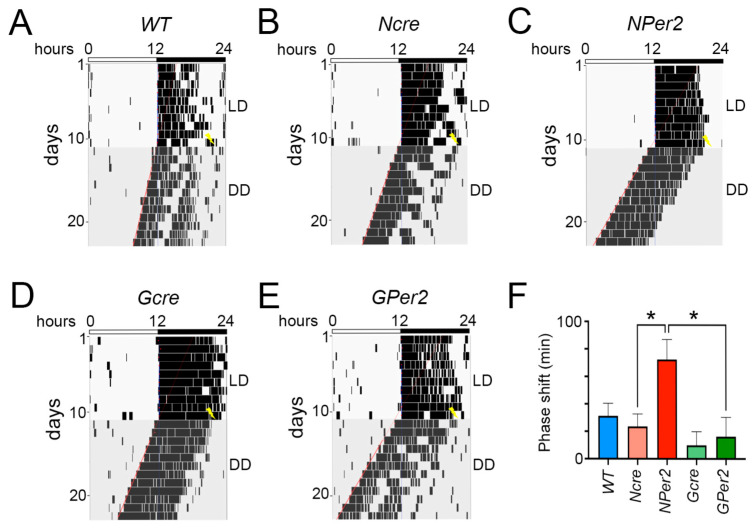
Representative actograms of wheel-running locomotor activity from mice receiving a light pulse (LP) at ZT22. (**A**) Wild-type (*WT*), (**B**) nestin-cre (*Ncre*) control animals, (**C**) neuronal *Per2* KO (*NPer2*) mice, (**D**) gfap-cre (*Gcre*) control animals, and (**E**) glial *Per2* KO (*GPer2*) mice. Mice were kept in a light/dark cycle (LD; 12:12 h) and received an LP of 15 min duration at ZT22 (yellow flash). Subsequently, they were released into constant darkness (DD). White and black bars on top of each actogram indicate light and dark phases, respectively. The red lines indicate activity onset in DD. The blue lines represent activity onset in LD condition. (**F**) Quantification of phase shifts after an LP at ZT22, with the following number of animals: *WT* and *Gcre*: *n* = 11 animals/genotype, and *Ncre*, *NPer2* and *GPer2*: *n* = 8 animals/genotype. Data are presented as mean ± SEM. One-way ANOVA: * *p* < 0.05 indicates differences between genotypes.

When LP was applied at ZT22 followed by DD (Figure 6), the *WT* and *Ncre* groups exhibited approximately 30 min phase advance (31.4 ± 9.1 min and 23.63 ± 9.1 min, respectively) and were not significantly different (*p* > 0.05), whereas *NPer2* revealed a significantly longer phase advance (72.4 ± 14.6 min) compared to its control, *Ncre* (*p* < 0.05). On the other hand, mice from Gcre and *GPer2* could not display a distinct phase advance (9.91 ± 10 min and 16.3 ± 14.1 min, respectively). No significant differences were observed between both groups as well as with the *WT* group (*p* > 0.05); however, *GPer2* showed a significant difference compared to *NPer2* (*p* < 0.05, Figure 6A–F).

**Figure 7:**
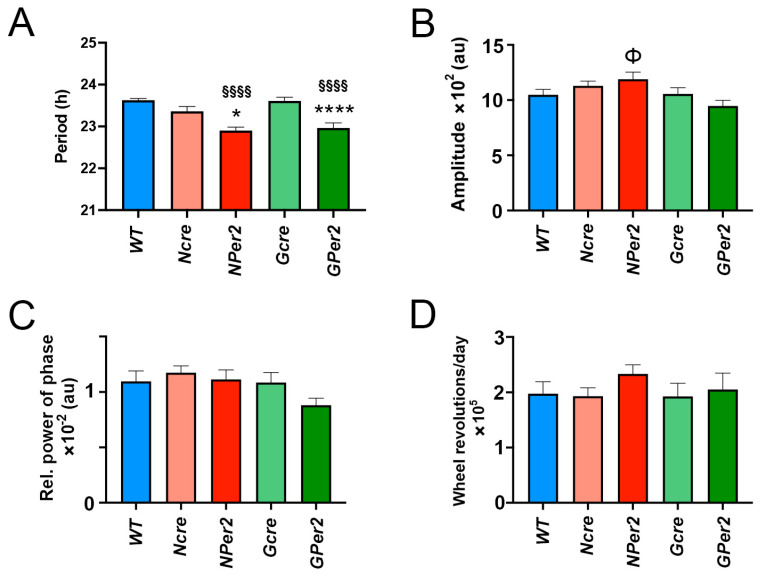
Effect of a light pulse (LP) at ZT22 on locomotor activity parameters. (**A**) Period length, (**B**) amplitude, (**C**) relative power of phase, and (**D**) total activity counts were analyzed in *WT* (blue), *Ncre* (rose), *NPer2* (red), *Gcre* (light green), and *GPer2* (green) animals in constant darkness (DD) after 15 min LP at ZT22. Data are presented as mean ± SEM, with *n* = 11 animals/genotype for *WT* and *Gcre*, and *n* = 8 animals/genotype for *Ncre*, *NPer2*, and *GPer2*. One-way ANOVA: ^§§§§^ *p* < 0.0001 indicates differences between *NPer2* and *GPer2*, for *WT* animals. * *p* < 0.05; **** *p* < 0.0001 indicate differences between *NPer2* and *GPer2* to their corresponding cre-control animals. ^Φ^ *p* < 0.05 indicates difference between *NPer2* and *GPer2*. Data are presented as mean ± SEM.

Additionally, after LP at ZT22, the three control groups (i.e., *WT*, *Ncre*, and *Gcre*) showed a non-significant shorter period length (23.6 h ± 0.04, 23.4 h ± 0.11, and 23.6 h ± 0.09, respectively; *p* > 0.05) (Figure 7A). Whereas KO groups (i.e., *NPer2* and *GPer2*) had significantly shorter period lengths (22.9 h ± 0.08 and 22.96 h ± 0.12) than their controls (*p* < 0.0001 and *p* < 0.05 *NPer2* with *WT* and *Ncre*, respectively, and *p* < 0.0001 *GPer2* with *WT* and *Gcre*). No difference was observed between KO groups (*p* > 0.05; Figure 7A). Moreover, *NPer2* exhibited a significantly higher amplitude than *GPer2* (*p* < 0.05, Figure 7B). However, LP was applied at ZT22 and did not affect the other locomotor activity parameters (i.e., FFT and total activity count) between different genotypes (*p* > 0.05; Figure 7C,D).

## 3. Discussion

In this study, we investigated the impact of *Per2* expression deficiency in astrocytes and neurons in mice on period and phase using wheel-running activity. Our findings revealed that the circadian period is shorter in mice lacking *Per2* in either astrocytes or neurons. Additionally, while light-induced phase shifts in mice deficient in astrocytic *Per2* were comparable to control animals, the absence of *Per2* in neurons significantly affected phase shifts.

The observed shorter period in *NPer2* and *GPer2* knockout (KO) mice suggests that both astrocytes and neurons contribute to circadian clock period regulation. This aligns with earlier studies demonstrating that circadian behavior is regulated not only by neurons [29] but also by astrocytes [18,19,22]. Prior research further highlighted that astrocytes sustain circadian oscillations and influence the circadian period in the SCN, albeit with lower efficacy than neurons [30]. Interestingly, while deletion of *Per2* in astrocytes shortens the behavioral period (Figure 3A), ablation of *Bmal1* in astrocytes lengthens it [18,23]. This reinforces the importance of astrocytic clock components in behavioral period regulation.

To examine phase shifts, we employed an Aschoff type II protocol and first assessed whether transitions from light/dark (LD) to constant darkness (DD) without a light pulse were consistent across genotypes. No significant differences were observed in activity onset post LD to DD transition among all groups of animals (Figure 2). This suggests that the shorter period observed in *NPer2* and *GPer2* mice (Figure 3A) did not significantly affect the phase shifts observed (Figs. 4 and 6). Moreover, total activity levels across genotypes showed no significant differences that could potentially impact activity onset following a light pulse (Figure 3D). However, all genotypes exhibited reduced amplitude and stability of circadian locomotor activity rhythm in DD (Figure 3B,C).

When light was applied at ZT14, all genotypes except *NPer2* mice displayed phase delays, with significantly reduced phase delays observed in *NPer2* animals (Figure 4). This phenotype mirrors that of mice with global *Per2* deficiency or *Per2* mutation leading to unstable protein [10,31]. Notably, mice lacking *Per2* in astrocytes (*GPer2* mice) exhibited normal phase delays, suggesting that astrocytic *Per2* does not influence phase delays, unlike neuronal *Per2* (Figure 4). This finding corroborates previous studies indicating that astrocytes do not regulate circadian phase in the SCN [30]. After a ZT14 light pulse, *NPer2* and *GPer2* mice showed shorter periods compared to controls (Figure 5), consistent with observations in the absence of light pulses (Figure 3). Similarly, other parameters, such as amplitude, relative power of phase, and total activity, showed no significant differences across genotypes (Figure 5), indicating these factors did not account for phenotypic variations in light-induced phase delays between *NPer2* and *GPer2* mice.

At ZT22, light induced behavioral phase advances in all genotypes (Figure 6). Interestingly, *NPer2* mice displayed significantly larger phase advances compared to all other genotypes, including *GPer2* mice. This phenotype parallels previous findings in mice with global *Per2* mutations, which showed greater phase advances relative to wild-type animals [10]. The enhanced phase advance in *NPer2* mice is unlikely to result from their shorter circadian period (Figure 7A), as *GPer2* mice also exhibit a shorter period but do not show increased phase advances (Figure 6F). This suggests that neuronal *Per2*, but not astrocytic *Per2*, influences phase advances—a conclusion consistent with observations of light-induced phase delays at ZT14 (Figure 4). These results further support the notion that astrocytes contribute to circadian period regulation but not phase modulation within the SCN [30].

When considering the results from the light pulses at ZT14 and ZT22 together, it is striking that mice lacking neuronal *Per2*—despite their impaired ability to delay the circadian clock—exhibit a disproportionately strong phase-advancing response to light during the late night. This suggests that neuronal Per2 plays a dual role—facilitating phase delays while concurrently restricting excessive phase advances.

One limitation of this study is the exclusive use of the Aschoff type II protocol, which does not exclude for potential influences of the preceding light/dark cycle prior to light pulse application and subsequent release into constant darkness. Future studies employing an Aschoff type I protocol in constant darkness could address this limitation.

In summary, our experiments shed light on the distinct roles of neuronal and astrocytic *Per2* in regulating rapid behavioral phase shifts caused by brief light pulses. We conclude that while astrocytic *Per2* contributes to circadian period regulation, neuronal *Per2* is involved in both period and phase modulation.

## 4. Materials and Methods

### 4.1. Housing of Mice and Mouse Strains

First, 2–4-month-old male mice were placed in completely light-isolated cabinets, previously described in [32], with a 12:12 h light/dark cycle (LD 7:19 h). Temperature (22 ± 2 °C monitored by temperature sensor, Technoline WS-9410, Berlin, Germany), humidity (40–50% monitored by humidity sensor, Technoline WS-9410, Berlin, Germany), and illumination (1000 Lux monitored by Luxmeter, Testo, GmbH & Co, Titisee-Neustadt, Germany) were stable in all cabinets. Mice were housed individually in cages (L: 280 mm × W: 105 mm × H: 125 mm) containing a running wheel (made of steel and 115 mm in diameter, Trixie GmbH, Tarp, Germany) and were provided with enrichments including small amounts of woodchip bedding, nestlet (5 × 5 cm), piece of carton, and open-sided tube as a type of refinement. Food and water were provided ad libitum.

The following mouse strains were used: glia-specific *GPer2* (*Per2*/*GfapCre*) knockout (KO) [13] and neuronal-specific *NPer2* (*Per2*/*NesCre*) KO [11] mice. Glial and neuronal *Per2* deletion was verified by genotyping, immunohistochemistry, and Western blot techniques in brain tissues of mice [11,13]. These animals were compared with their corresponding cre-line *GfapCre* (*Gcre*) [24], *NesCre* (*Ncre*) [25], and littermate wild-type (*WT*) mice as a control group. All experiments and procedures were performed according to the Schweizer Tierschutzgesetz guidelines and approved by the Canton of Fribourg and the cantonal commission for animal experiments (2022-32-FR, 35432).

### 4.2. Monitoring Circadian Locomotor Activity Rhythm

Circadian locomotor activity rhythm was evaluated using the running wheel. The running wheel revolutions were recorded by a magnetic circuit, previously described in [32,33], which is fixed vertically on the axis of the running wheel outside the cage. The circuit switches on and off according to the wheel’s revolution. These revolutions (indicating the locomotor activity) are captured in 1 min intervals by ClockLab 3 data acquisition system software (Acquisition Version 3.208, Analysis Version 6.0.36).

### 4.3. Experimental Design and Application of Light Pulse (Aschoff Type II Protocol)

The experimental design (Figure 1) was performed according to the Aschoff type II protocol [26,27]. For the 1st experimental condition, mice were kept in LD (12:12 h) for 2 weeks and locomotor activity was recorded until they had stable rhythms, then the mice were released into constant darkness (DD) without light pulse (LP) after the end of the last dark phase of the LD cycle by switching off the light in the cabinets using an automatic digital timer. Locomotor activity was recorded for at least 12 days. For the 2nd experimental condition, the mice readapted to the LD cycle for 2 weeks and were then released into DD with LP (using 2 visible light spectrum neon light sources: Lumilux cool daylight, 1000 Lux, 18 W, OSRAM, Munich, Germany) at ZT14 (early night) for 15 min on the last LD night, and again locomotor activity was recorded for at least 12 days. For the 3rd experimental condition, the previous step was repeated but with an LP at ZT22 (late night) instead of ZT14. During the constant darkness (DD) periods, mice were checked under dim-red light to control the water and food levels as well as their health status.

### 4.4. Analysis of Circadian Locomotor Activity Rhythm Parameters

Phase shift (i.e., phase resetting) in DD with/without LP as well as other circadian locomotor activity rhythm parameters (e.g., actograms, period length, amplitude, total locomotor activity count, and relative power of phase (FFT)) were evaluated using ClockLab analysis (Actimetrics) software version 6.0.36.

To analyze the phase shift in each condition, a fitting line was set through 7 consecutive activity LD onsets before the transition into DD with/without LP. Another fitting line was set through at least 10 consecutive activity DD onsets after the transition into DD with/without LP (the first two days after the transition phase were excluded from the calculations). The difference between the two fitting lines on the base of the day that follows the transition to the DD with/without LP is considered the phase shift value [33].

For other circadian locomotor activity rhythm parameters, 7 consecutive activity onsets before and after the transition into DD with/without LP were analyzed (the first two days after the transition phase were excluded from the calculations).

### 4.5. Statistical Analysis

GraphPad Prism software (version 10.3.1) was used for statistical analysis. After performing normality tests, parametric paired t-test was used to determine the significant difference before and after the transition phase in each group. In addition, the significant difference between three groups or more was performed using parametric one-way ANOVA followed by Dunnett’s test for multiple comparisons. Data are presented as mean ± SEM and are considered significant when the *p* value < 0.05.

The raw data appear in Appendix A.

## Data Availability

The original contributions presented in this study are included in the article. Further inquiries can be directed to the corresponding author.

## Appendix A. Raw Data

**No LP**

Amplitude DD

**WT**	***Ncre***	***NPer2***	***Gcre***	***GPer2***
1048.53	1166.66	873.76	847.05	896.51
1107.46	939.45	1150.14	1130.42	1519.94
769.1	1172.49		755.9	820.66
941.43	1431.96	1179.41	1154.85	1331.63
1120.08	1032.02	1246.67	1046.32	578.5
946.5	1088.77	1483.93	1083.5	956.04
856.11	1076.84	857.01	863.19	1008.89
713.92	1116.14	1086.83	654.01	821.58
1419.01	1029.68		1207.47	1385.45
834.72	1176.51	1033.58	1205.88	1003.66
636.1	1247.61		863.43	938.92

Period DD

**WT**	***Ncre***	***NPer2***	***Gcre***	***GPer2***
24	23.6	23	23.5	23.1
23.7	23.9	23.1	23.6	23.3
23.5	23.6		23.7	22.9
23.9	23.3	23.2	23.4	22.8
23.7	23.5	22.8	23.6	22.8
24	23.5	22.8	23.2	23.3
23.7	23	23.3	23.8	23.1
23.7	23	23.1	24	23.1
23.7	23.8		23.9	23.2
23.9	23.6	22.8	24	23
24	23.6		23.9	23.8

Total activity DD

**WT**	***Ncre***	***NPer2***	***Gcre***	***GPer2***
101,776	102,147	156,830	310,204	198,360
243,756	86,527	239,398	252,087	311,413
159,279	159,432		132,978	157,477
174,192	189,439	192,013	260,508	268,829
298,636	152,363	211,167	202,403	98,091
215,897	190,575	195,320	234,633	175,981
147,788	175,678	187,758	119,607	198,859
91,452	215,875	142,819	53,304	66,796
130,728	147,310		183,875	185,661
227,335	153,652	206,793	187,674	168,546
99,150	175,600		106,857	165,544

FFT DD

**WT**	***Ncre***	***NPer2***	***Gcre***	***GPer2***
0.0067	0.01	0.0072	0.0121	0.0083
0.0111	0.0086	0.0098	0.0113	0.0176
0.0094	0.0138		0.0086	0.0064
0.0126	0.013	0.0132	0.014	0.0119
0.0142	0.0098	0.0104	0.0111	0.0043
0.0112	0.0108	0.0122	0.0129	0.0103
0.0094	0.0104	0.012	0.0084	0.01
0.0074	0.011	0.0086	0.003	0.006
0.0141	0.014		0.0112	0.0138
0.0099	0.0113	0.0095	0.0139	0.0085
0.0063	0.0139		0.0082	0.0104

**LP ZT14**

Amplitude

**WT**	***Ncre***	***NPer2***	***Gcre***	***GPer2***
1019.43	1300.7	1220.39	1013.35	932.12
1345.37	1402.63	1049.52	1028.3	1078.58
1389.67	1410.13	1252	1194.22	1399.02
637.11	1167.43	1396.98	1097.29	1397.94
1027.31	1365.22	1420.78	1065.1	1289.94
696.98	1238.05	1157.23	954.04	420.27
1175.41	1277.42	802.75	661.55	917.82
955.56	1352.86	1108.35	1463.77	830.01
1119.89			913.79	
866.17			1253.59	
1245.74			854.44	

Period

**WT**	***Ncre***	***NPer2***	***Gcre***	***GPer2***
24	23.7	23.2	24	23.7
23.8	23.7	23.5	23.8	23.3
23.9	23.5	23.4	23.7	23
23.9	23.4	23.5	23.9	23.6
23.7	23.7	23.2	23.8	23.3
23.9	23.6	23.4	23.8	23.6
24	23.4	23.5	23.9	23.8
23.7	23.7	22.8	23.6	23.2
23.7			24	
23.8			23.8	
23.5			23.9	

Total activity

**WT**	***Ncre***	***NPer2***	***Gcre***	***GPer2***
142,606	224,604	247,751	357,153	252,482
324,977	303,940	240,917	383,277	220,009
280,846	227,242	200,184	238,549	246,312
233,007	261,473	238,985	282,009	302,316
323,706	333,332	244,618	259,068	283,594
198,528	200,523	224,232	270,496	156,655
277,119	288,343	277,498	223,198	244,194
161,037	261,195	359,083	140,379	170,584
161,645			193,362	227,513
125,330			249,221	
286,006			175,797	

FFT

**WT**	***Ncre***	***NPer2***	***Gcre***	***GPer2***
0.0137	0.0128	0.0109	0.0137	0.0103
0.0131	0.0175	0.011	0.0104	0.0114
0.0143	0.0129	0.0165	0.0142	0.0126
0.0129	0.012	0.0139	0.0119	0.0088
0.0108	0.0175	0.0135	0.0133	0.0117
0.0107	0.0124	0.0081	0.0132	0.0091
0.0093	0.0138	0.0128	0.0109	0.008
0.014	0.0158	0.0133	0.0142	0.011
0.0118			0.0071	
0.01			0.0145	
0.0136			0.0108	

Phase shift ZT14

**WT**	***Ncre***	***NPer2***	***Gcre***	***GPer2***
−76	−90	11	−87	−48
−39	−11	−5	−61	−59
−29	−65	13	−66	−85
−26	−49	−43	−77	−116
−65	−24	−4	−17	−46
−48	−105	−50	−29	−104
−87	−62	5	−32	−62
−60	−35	−51	−29	−61
−88			−50	
−43			−50	
−72			−102	

**LP ZT22**

Amplitude

**WT**	***Ncre***	***NPer2***	***Gcre***	***GPer2***
1113.27	1134.13	995.12	1120.35	1092.78
940.32	1101.71	1217.89	1233.17	1074.73
1232.98	1221.09	1323.06	1215.42	1042.41
1254.89	1325.46	1168.04	1336.93	790.86
1161.42	1103.02	1560.8	1081.62	1067.53
1182.5	1194.01	1009.29	1136.66	762.46
992.25	1015.77	1090.77	885.37	774.85
1096.79	939.48	1142.46	687.93	964.04
806.13			963.49	
761.89			884.6	
979.92			1065.84	

Period

**WT**	***Ncre***	***NPer2***	***Gcre***	***GPer2***
23.9	23.5	22.5	23.4	22.7
23.4	23.8	23.1	23.6	22.4
23.6	23.7	23.2	23.5	23
23.5	23	22.8	23.4	23.5
23.7	23.5	22.8	23.1	23.2
23.6	23.4	23	23.6	22.7
23.6	23	23.1	23.7	23.1
23.5	23	22.7	24.2	23.1
23.7			23.5	
23.7			24	
23.7			23.7	

FFT

**WT**	***Ncre***	***NPer2***	***Gcre***	***GPer2***
0.0082	0.0113	0.0063	0.0117	0.0115
0.0119	0.01	0.0114	0.0128	0.0083
0.0105	0.0154	0.0138	0.0129	0.0109
0.0144	0.0112	0.0117	0.0126	0.006
0.0148	0.0111	0.014	0.013	0.0097
0.0125	0.0132	0.0118	0.0119	0.0081
0.0108	0.0103	0.0099	0.0112	0.0076
0.0144	0.0114	0.0102	0.0037	0.0084
0.0091			0.0087	
0.0044			0.0075	
0.0096			0.0134	

Total activity

**WT**	***Ncre***	***NPer2***	***Gcre***	***GPer2***
147,100	149,079	247,655	303,918	302,347
297,495	119,340	271,843	286,338	240,363
210,048	236,449	214,717	181,117	340,955
328,076	201,126	196,570	269369	98,882
194,285	206,970	260,356	245,470	162,140
263,782	237,568	235,270	192,252	141,856
156,855	168,764	147,220	122,239	204,869
168,960	224,490	293,542	70,730	149,232
163,609			108,370	
91,811			118,755	
150,743			220,608	

Phase shift

**WT**	***Ncre***	***NPer2***	***Gcre***	***GPer2***
37	35	36	8	−37
15	22	160	32	−1
41	16	51	−9	−6
12	32	77	6	29
14	31	40	−58	36
22	3	43	32	−25
24	−19	76	−9	73
32	69	96	68	61
117			−10	
20			10	
11			39	
